# Supplementary material for: Polyketide synthase gene clusters in the ericoid mycorrhizal fungus Oidiodendron maius: genome-wide prediction, expression profiling, and functional characterization of knockout mutants
Source: Front Plant Sci. 2026 Sep 16;17:1924430. doi: 10.3389/fpls.2026.1924430 (PMC13623903; doi:10.3389/fpls.2026.1924430)
Supplement: Supplementary file 1 [file SupplementaryFile1.zip › Supplementary Materials/Supplementary Folder 1.zip!/Oidma1_AssemblyScaffolds_Repeatmasked/knownclusterblast/cluster44/jgi.p_Oidma1_45558_mibig_hits.html]

| MIBiG Protein | Description | MIBiG Cluster | MiBiG Product | % ID | % Coverage | BLAST Score | E-value |
| --- | --- | --- | --- | --- | --- | --- | --- |
| CDM31314.1 | Short-chain\_dehydrogenase/reductase\_SDR | BGC0000667 | PR\_toxin\_biosynthetic\_gene\_cluster | 37 | 90.4761904762 | 180 | 2.5e-45 |
| BAO66515.1 | retinol\_dehydrogenase | BGC0000042 | Cremimycin\_biosynthetic\_gene\_cluster | 33 | 80.0 | 118 | 2e-26 |
| BAR73004.1 | putative\_short-chain\_dehydrogenase | BGC0001194 | Hitachimycin\_biosynthetic\_gene\_cluster | 34 | 64.126984127 | 110 | 4.1e-24 |
| AAL06687.1 | oxidoreductase | BGC0000965 | C-1027\_biosynthetic\_gene\_cluster | 35 | 63.8095238095 | 102 | 8.6e-22 |
| ALU98449.1 | short-chain\_dehydrogenase | BGC0001397 | Lidamycin\_biosynthetic\_gene\_cluster | 35 | 63.8095238095 | 102 | 8.6e-22 |
| BAQ25463.1 | short-chain\_dehydrogenase | BGC0001280 | Betaenone\_C\_/\_betaenone\_A\_biosynthetic\_gene\_cluster | 27 | 69.2063492063 | 74 | 3.3e-13 |
| ABY83178.1 | Azi40 | BGC0000960 | Azinomycin\_B\_biosynthetic\_gene\_cluster | 24 | 68.8888888889 | 56 | 5.4e-08 |
| AJW65399.1 | oxidoreductase | BGC0001195 | Nocardiopsin\_biosynthetic\_gene\_cluster | 27 | 43.8095238095 | 50 | 3.9e-06 |
